# Investigating the microstructure of plant leaves in 3D with lab-based X-ray computed tomography

**DOI:** 10.1186/s13007-018-0367-7

**Published:** 2018-11-12

**Authors:** Andrew W. Mathers, Christopher Hepworth, Alice L. Baillie, Jen Sloan, Hannah Jones, Marjorie Lundgren, Andrew J. Fleming, Sacha J. Mooney, Craig J. Sturrock

**Affiliations:** 1 Division of Agricultural and Environmental Sciences, School of Biosciences, University of Nottingham, Sutton Bonington Campus, Loughborough, Leicestershire LE12 5RD UK; 2 Department of Animal and Plant Sciences, University of Sheffield, Western Bank, Sheffield, S10 2TN UK; 3 Lancaster Environment Centre, Lancaster University, LEC 2 Yellow Zone B43, Lancaster, LA1 4YQ UK

**Keywords:** X-ray computed tomography (microCT), Porosity, Leaf structure, Air channels, Gas exchange, Photosynthesis

## Abstract

**Background:**

Leaf cellular architecture plays an important role in setting limits for carbon assimilation and, thus, photosynthetic performance. However, the low density, fine structure, and sensitivity to desiccation of plant tissue has presented challenges to its quantification. Classical methods of tissue fixation and embedding prior to 2D microscopy of sections is both laborious and susceptible to artefacts that can skew the values obtained. Here we report an image analysis pipeline that provides quantitative descriptors of plant leaf intercellular airspace using lab-based X-ray computed tomography (microCT). We demonstrate successful visualisation and quantification of differences in leaf intercellular airspace in 3D for a range of species (including both dicots and monocots) and provide a comparison with a standard 2D analysis of leaf sections.

**Results:**

We used the microCT image pipeline to obtain estimates of leaf porosity and mesophyll exposed surface area (S_mes_) for three dicot species (Arabidopsis, tomato and pea) and three monocot grasses (barley, oat and rice). The imaging pipeline consisted of (1) a masking operation to remove the background airspace surrounding the leaf, (2) segmentation by an automated threshold in ImageJ and then (3) quantification of the extracted pores using the ImageJ ‘Analyze Particles’ tool. Arabidopsis had the highest porosity and lowest S_mes_ for the dicot species whereas barley had the highest porosity and the highest S_mes_ for the grass species. Comparison of porosity and S_mes_ estimates from 3D microCT analysis and 2D analysis of sections indicates that both methods provide a comparable estimate of porosity but the 2D method may underestimate S_mes_ by almost 50%. A deeper study of porosity revealed similarities and differences in the asymmetric distribution of airspace between the species analysed.

**Conclusions:**

Our results demonstrate the utility of high resolution imaging of leaf intercellular airspace networks by lab-based microCT and provide quantitative data on descriptors of leaf cellular architecture. They indicate there is a range of porosity and S_mes_ values in different species and that there is not a simple relationship between these parameters, suggesting the importance of cell size, shape and packing in the determination of cellular parameters proposed to influence leaf photosynthetic performance.

**Electronic supplementary material:**

The online version of this article (10.1186/s13007-018-0367-7) contains supplementary material, which is available to authorized users.

## Background

It is estimated that a doubling in agricultural productivity will be required over the next three decades to meet the increasing food demand of a rapidly growing global population [1]. Photosynthesis is an important driver of food production but has thus far been little improved by crop breeding or engineering [2]. Although significant advances have recently begin to be reported via engineering photosynthetic biochemistry [3–5], less progress has been made in the optimisation of internal leaf architecture (the arrangement of cells and airspaces within the leaf) which is also thought to limit photosynthetic carbon assimilation [6].

For example, the surface area of mesophyll cells exposed to intercellular airspaces (*S*_*mes*_) has been shown to be positively correlated with photosynthetic performance [7, 8] presumably by facilitating increased diffusional flux of CO_2_. A clearer understanding of how leaf architectural traits, such as *S*_*mes*_ and porosity (the proportion of the leaf volume occupied by airspace), influence photosynthetic potential is vital if we are to successfully optimise leaf cellular architecture to maximise carbon assimilation.

However, imaging the microstructure of plant leaves can be challenging due to their low density, fine structure, and sensitivity to desiccation. A number of established stereological approaches are commonly used to quantify leaf structural parameters, such as *S*_*mes*_, from two-dimensional (2D) tissue cross-sections of chemically fixed, resin embedded tissue. Measurements of lengths or areas from the cross-sectional images are transformed using correction factors to generate estimations of three-dimensional (3D) geometry [9–11]. However these techniques are destructive, labour intensive, and in some cases the process of tissue preparation can alter the parameters being measured (e.g. poorly sectioned samples or sectioned at oblique angles), potentially leading to underestimation of values for *S*_*mes*_ by as much as 30% [12].

More recently, 3D imaging techniques have been applied to simplify and improve quantification of plant structures. Tomographic techniques generate non-destructive serial section images through the sample of interest. A range of tomography-based techniques is now available for imaging of low density materials, the majority of which were developed in medical physics as non-invasive diagnostic tools. For example, nuclear magnetic resonance imaging (MRI) allows visualisation of materials based on their water content. It has been successfully applied to studies of seedling germination, plant root growth and architecture in soils [13–18], but its relatively coarse spatial resolution (> 50 μm) makes it unsuitable for imaging the fine microstructure of aerial plant tissues. Positron emission tomography (PET) uses short-lived radioisotopes (typically ^11^C and ^14^C) to determine the assimilation of compounds in living organisms with exceptionally high sensitivity (picomolar order of magnitude). However, the spatial resolution of PET is even more coarse than MRI (1–5 mm), so structural information must be gathered independently [19]. Optical projection tomography (OPT) uses visible light (photons) to discriminate between materials. Whilst OPT is capable of capturing high resolution images (ca. 5 μm), like other optical techniques it is limited by sample thickness and requires chemical fixation and staining of tissues [20] which can often be time consuming and place limitations on throughput. This technique is, however, useful for studying the spatial distribution of marker gene expression in stained plant tissues, as demonstrated by Lee et al. [21]. Finally, X-ray computed microtomography (microCT) combines the advantages of high resolution and excellent depth penetration by using X-rays to visualise structure. The X-ray attenuation coefficient of a material is dictated by its density and atomic number [22], so the technique is capable of imaging plant tissue structures by discriminating low density intercellular airspaces from denser cellular material [23].

MicroCT can be conducted in synchrotron facilities or using more compact, lab-based equipment. Synchrotron-based microCT (SRXCT) has the advantage of using a high flux, coherent, monochromatic photon beam permitting collection of both absorption and phase contrast radiographic images at high resolution (e.g. 0.35–5 μm image pixel size. TOMCAT Beamline, Swiss Light Source [24]). This technique has been successfully used to discriminate individual plant cells and to investigate airspace connectivity in fresh fruit tissue [25] and leaves [12]. However, the expense and scarcity of such facilities limits the use of synchrotron-based microCT. Although lab-based microCT systems can now achieve similar resolution range to SRXCT, the greater accessibility of lab based equipment has allowed it to be used to study many plant structural features such as trichome distribution on *Arabidopsis* leaves [26], leaf venation [27], panicle development and seed density in rice [28], floral shape variation in orchids [29] and volume and surface area measurements of inflorescences of tulips and proteaceae [30]. As benchtop microCT systems have a lower X-ray flux, sample damage is generally considered to be lower compared to Synchrotron based systems. It is therefore possible to perform non-destructive imaging of live plants, allowing repeat measurements on the same individuals over time or before and after a treatment. However, Dhondt et al. [23] reported inhibition of seedling growth after multiple rounds of scanning, suggesting that there is a limit to the intensity and/or frequency of scanning of live tissue that is possible without affecting development. In microCT systems, the low level of X-ray absorption by plant tissue presents challenges to differentiate cellular level structures such as individual cell types due to insufficient image contrast. This can be overcome to some extent by the use of low energies. The application of contrast agent solutions (e.g. iodine, gadolinium, barium) have also provided promising results to overcome this issue [23, 31], but the use of contrast agents do not appear to increase image quality in all systems [32], and can lead to longer preparation times compared to scanning fresh tissue.

Several studies have used the image data generated by microCT to calculate quantitative descriptors of plant tissue structure. For example Schneider et al. [27] used microCT images to calculate vein density in leaf tissue, and Herremans et al. [33] conducted a very detailed analysis of fruit tissue structure. We have recently used microCT to quantify leaf cellular architecture of Arabidopsis mutants, uncovering relationships between structural parameters and photosynthetic performance [34, 35]. Here, we demonstrate that lab-based X-ray microCT can be used to visualise and quantify differences in leaf intercellular airspace in 3D in a range of species including both dicots and monocots. Our method yields high resolution images (ca. 2.5–2.75 μm) and does not require laborious chemical fixation or staining techniques to prepare the samples. An image analysis pipeline has been developed to provide quantitative descriptors of plant leaf intercellular airspace. We have focused on leaf porosity and *S*_*mes*_, but methods for further structural analyses are included in the Additional file 1. These 3D measurements provide insight into the available pathways for gas flow within the leaf, which in turn influences the potential photosynthetic productivity of the plant.

## Methods

### Plant growth

Arabidopsis seeds (Col-0 ecotype) were sown directly into 60 × 60 × 80 mm pots of damp, lightly compressed soil (3:1 Levington M3 compost:perlite) and stratified at 4 °C for 7 days before transfer into a controlled environment chamber (Conviron, Canada) under short day conditions (12 h light 22 °C/12 h dark 15 °C, 200 µmol m^−2^ s^−1^ PAR at rosette level, 60% humidity). Leaf discs were excised from the largest leaves for scanning 30 days after germination. Tomato (*Solanum lycopersicum* var. Ailsa Craig), pea (*Pisum sativum* var. Arvense), barley (*Hordeum vulgare* var. Tipple Fulbourn) and oat (*Avena sativa*), were sown in 20 × 20 × 30 cm pots of M3 compost and grown under long day conditions (16 h light 22 °C/8 h dark 15 °C, 200 µmol m^−2^ s^−1^ PAR, 60% humidity). Leaf discs were excised from the largest, mature leaves for scanning. Rice seeds (*Oryza latifolia*) were germinated on wet filter paper in 90 mm diameter, 20 mm deep petri plates, and transplanted into water-saturated soil (70% v/v Kettering Loam (Boughton, UK), 23% v/v Vitax John Innes No. 3 (Leicester, UK), 5% v/v silica sand and 2% v/v Osmocote Extract Standard 5–6 month slow release fertiliser (Ipswich, UK)) in 105 × 105 × 185 mm pots, 8 days after germination. Rice plants had a constant water supply from the pot base and were grown in a controlled environment chamber (Conviron, Canada) with 12 h, 30 °C days and 12 h 24 °C nights, 700 µmol m^−2^ s^−1^ PAR at canopy level and 60% relative humidity.

For rice and Arabidopsis, n = 5. For the other species, and for the 2D analysis of rice, leaf sections n = 4. To allow comparison of leaves of the same species (or mutants) we selected leaf 5 for analysis in our experimental studies so they are at same developmental growth stage.

### Sample preparation for microCT

**Fig. 1:**
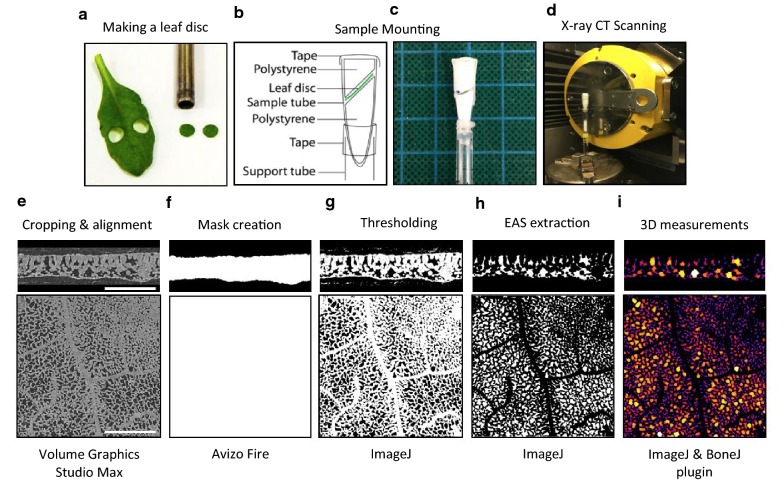
Step-by-step stages of the microCT workflow. **a** Excising the leaf discs, **b** sample mounting schematic, **c** image of mounted sample, **d** X-ray CT scanning and **e**–**i** image analysis workflow for extracting intercellular airspace from plant leaf scans, illustrated using both side-on (ZY orientation, top row) and top-down (XY orientation, palisade mesophyll layer, bottom row) views of an *Arabidopsis thaliana* leaf selection (400 × 400 pixels). Resolution = 2.75 µm. Scale bars = 0.4 mm

Single leaf discs (5 mm diameter) were excised from the mid-point (length-ways) of selected leaves using a stainless steel cork borer and avoiding the mid-vein (Fig. 1a). Leaf discs were mounted between low density polystyrene, at a 45° angle to reduce the number of angular projections through the maximum thickness of the sample, in 1.5 mL polypropylene micro centrifuge tubes, mounted on a 10 cm length of a plastic pipettes (Fig. 1b–d). Sample holder components were selected based on their rigidity, providing a tight fit to reduce sample movement, and low X-ray absorption, enabling good contrast with leaf material. Sample holders were sealed with Sellotape^®^ to reduce desiccation and acclimatised for 5 min with the sample in the X-ray beam. Leaf discs from monocot species were positioned so that the veins were parallel to the X-ray source prior to scanning to aid alignment after reconstruction.

### X-ray microCT scanning

Single microCT scans of leaf discs were performed using a GE Phoenix Nanotom S 180NF (GE Sensing and Inspection Technologies GmbH, Wunstorf, Germany) fitted with a tungsten transmission target and a 5 MP (2304 × 2304 pixel) CMOS digital detector (Hamamatsu Photonics KK, Shizuoka, Japan). A three-point detector calibration was performed, collecting an average of 100 images, with 10 skip images per gain point. Scans were obtained at a spatial resolution of 2.75 μm (2304 × 1400 pixel field of view), with an electron acceleration energy of 85 kV and a current of 100 μA (higher spatial resolutions are possible if a smaller diameter sample can be used). Detector exposure time was 500 ms, collecting 3600 projections in ‘fast scan’ mode (sample rotates continuously), with no averaging or skip images and no pixel binning (1 × 1), resulting in a scan duration of 30 min per sample.

### Reconstruction

Radiograph reconstruction was carried out using Phoenix Datos|x rec 2 reconstruction software (version 2.3.3; GE Sensing and Inspection Technologies GmbH, Wunstorf, Germany). Radiographs were assessed for sample movement using the autoscan|optimiser module, by comparing the difference between the first and last projection image (0° and 360° rotation) and applying an automatic directional and/or scale correction if movement and/or shrinkage were apparent. Any sample that required more than 3 pixel shift in x or y axis were either rescanned or disregarded as the image quality in these images was low. Beam hardening artefacts were mitigated using the multiple materials function in the BHC + module. A beam hardening correction of 7 was determined to be the most appropriate for plant leaves. Finally, radiographs were manually cropped i.e. resized to remove the scanned area beyond the leaf sample before being reconstructed into 3D volumes using a filtered back-projection algorithm.

### Image analysis

An illustration of the image analysis workflow is provided in Fig. 1e–i.

#### Alignment and cropping

Grayscale volumes were aligned in 3D (adaxial leaf surface facing up), cropped to remove any damaged leaf material at the disc periphery, and converted to stacks of TIFF images in the Z dimension using VG StudioMAX (version 2.2.0; Volume Graphics GmbH, Heidelberg, Germany).

#### Mask creation

Leaf discs were segmented from the surrounding sample holder by creating material masks in Avizo Fire software (version 6.0.0 Fire; Thermo Fisher Scientific, USA), using the ‘Label Field’ function and then binarising the selection.

#### Thresholding

Individual grayscale TIFF stacks were thresholded using the ‘Threshold’ function in the open source software package ImageJ (version 1.48; [36]) and saved as binary TIFF stacks, differentiating solid material from airspace. The automated thresholding algorithm was selected based on comparison between the binarised and the greyscale images, to account for small differences between scans in sample/background contrast, leaf water content and polystyrene elements. Previous research by our group has shown that the *IJ Iso*-*data* algorithm proved effective for thresholding Arabidopsis [35]. However, it should be highlighted that a range of automated thresholding algorithms are available within ImageJ and will result in different outputs depending on the grayscale distributions of the image. This unfortunately, results in some level of manual selection of the most appropriate threshold algorithm. We would strongly recommend that the same threshold algorithm is used for all samples within the same study. For the rice and cereal leaves, the Li algorithm was used as they presented a finer pore structure. Material masks were thresholded using the automatic thresholding method ‘MaxEntropy’. All thresholded images were saved as binary TIFF stacks.

#### Intercellular airspace extraction

Binary material masks were combined with thresholded image stacks using the ‘Image Calculator’ function in ImageJ to create a composite image stack, isolating the extracellular airspace within each leaf disc.

#### Noise removal

Scans were de-noised using the ‘Remove Outliers’ function in ImageJ. Foreground and background particles < 3 × the spatial resolution were removed.

#### Region of interest selection

The inclusion of the mid-rib and/or major veins in images subjected to 3D analysis can artificially increase porosity measurements. In monocots, where vasculature is arranged in parallel cell files, regions of interest were selected between major veins. In rice in particular, which has dense vasculature, three 200 × 200 voxel regions were selected for analysis, and all 3D measurements were averaged across these technical replicates to provide representative data for the leaf disc as a whole. In all other species a region of interest (ROI) of ≥ 400 × 400 voxels was used. Due to the non-uniform structure and irregular vasculature of dicot leaves, it was not possible to entirely exclude vasculature, but the largest veins were avoided.

### 3D measurements

All 3D measurements were conducted using ImageJ (version 1.48; [36]). Leaf disc porosity, the number of individual air channels, the porosity distribution through the leaf disc depth, and the surface area of mesophyll cells exposed to intercellular airspace (*S*_*mes*_) were all calculated from data acquired using the ImageJ function ‘Analyze Particles’. Leaf porosity (%) was calculated using Eq. 1:

1 $$Porosity = \left( {\frac{{\sum A_{p} }}{{\sum A_{m} }}} \right) \times 100$$

where, *ΣA*_p_ and *ΣA*_m_ are the summation of the area (mm^2^) occupied by pores and the area of the mask for all slices within the entire z-stack. The distribution of porosity throughout the leaf disc was plotted by calculation of porosity on a slice-by-slice basis (increments equal to individual slice thickness, which is determined by the CT scan resolution) in the Z dimension, and plotted from the adaxial to abaxial surface.

*S*_*mes*_ (mm^2^ mm^−2^) was calculated using Eq. 2:

2 $$S_{\text{mes}} = \frac{{\sum P_{p} \times RES}}{{\sum A_{m} }}$$

where, *ΣP*_p_ is the summation of the perimeters (mm) of each individual pore present within the entire z-stack and *RES* is the spatial resolution of the CT scan (mm). The number of individual pores, and their perimeters, were direct outputs of the ‘Analyze Particles’ function. The perimeter measure is implemented within the PolygonRoi class and is calculated by accounting for the straight and corner pixels of the boundary. In brief, straight edge pixels are measured as length 1, with corner pixels length $\sqrt 2$.

Representative 3D renderings of plant material, with air channel diameters illustrated by heat map, were constructed in VG StudioMAX (version 2.2.0; Volume Graphics GmbH, Heidelberg, Germany) using the isosurface and Phong rendering tools. The heat map data was an output of the ‘Thickness’ function in the ImageJ plugin BoneJ (version 1.3.14; [37]) which also provides the mean and maximum channel diameter for each stack.

### Sample preparation for 2D analysis of fixed tissue sections

Leaf discs of *O. latifolia* were fixed in 4% v/v formaldehyde in PEM buffer (1.5% w/v Pipes, 0.19% w/v EGTA, 0.124% w/v MgSO_4_, pH 7) immediately after CT scanning. After no more than 72 h, samples were rinsed in PEM buffer three times for 10 min each. Samples were dehydrated in an ascending ethanol series (10%, 30%, 50%, 70%, 90%, 100% v/v ethanol, 1 h each) then infiltrated with an ascending series of LR white resin (London Resin Company) in ethanol (10%, 20%, 30%, 50%, 70%, 90% v/v 1 h each then 3 × 8 + hours in 100% resin). Samples were kept at 4 °C throughout dehydration and infiltration. Finally samples were stood vertically in gelatine capsules filled with resin and left to polymerise for 5 days at 37 °C. 2 µm sections were cut with a Reichert-Jung Ultracut E ultramicrotome and dried onto vectabond-coated multi-well slides. 4–5 sections were imaged per biological replicate, each of which was at least a cell’s length apart. Sections were stained for 5 min in a 0.1 mg mL^−1^ solution of propidium iodide in water and rinsed in water before imaging. Samples were imaged using a Leica DM6 microscope and camera equipped with a CoolLED fluorescence system, and images were captured using LASX software. Samples were illuminated with the 535 nm LED line, and visualised through the Y3 filter.

### 2D measurements

The workflow for stereological analysis is illustrated in Additional file 2: Fig. S1. Masks representing total leaf area (Additional file 2: Fig. S1B) and individual airspaces (Additional file 2: Fig. S1C) were generated using ImageJ (FIJI v1.51u; [38] with the connection thresholding and edge detection plugins). Masks were smoothed using the Median filter, with a radius of 3 pixels. Airspace area was expressed as a percentage of total leaf area to give an estimate of porosity (the fraction of leaf volume occupied by intercellular airspace).

The perimeter of each individually segmented airspace was measured (Additional file 2: Fig. S1D) and summed to give the total perimeter of pores exposed to intercellular airspace (*∑P*_*p*_, mm). The width of the microscope section analysed (W, mm) was measured (Additional file 2: Fig. S1A). The total cell surface area exposed to intercellular airspace per leaf surface area (*S*_*mes*_, mm^2^ mm^−2^) was calculated using the Eq. 3.

3 $$S_{mes} = \frac{{\sum P_{p} }}{W} \times F$$

where F is a stereological correction factor. In order to estimate 3D *S*_*mes*_ from this data, airspaces were assumed to have a general prolate spheroid shape with the major axis being twice the length of the other two minor axes, as in Giuliani et al. [39], and accordingly, based on Thain [10], an *F* value of 1.42 was used.

### Statistical analyses

All statistical analyses were conducted in Graphpad Prism software (version 7.03).

## Results

### 3D analysis of leaves from common dicot and monocot reference species

**Fig. 2:**
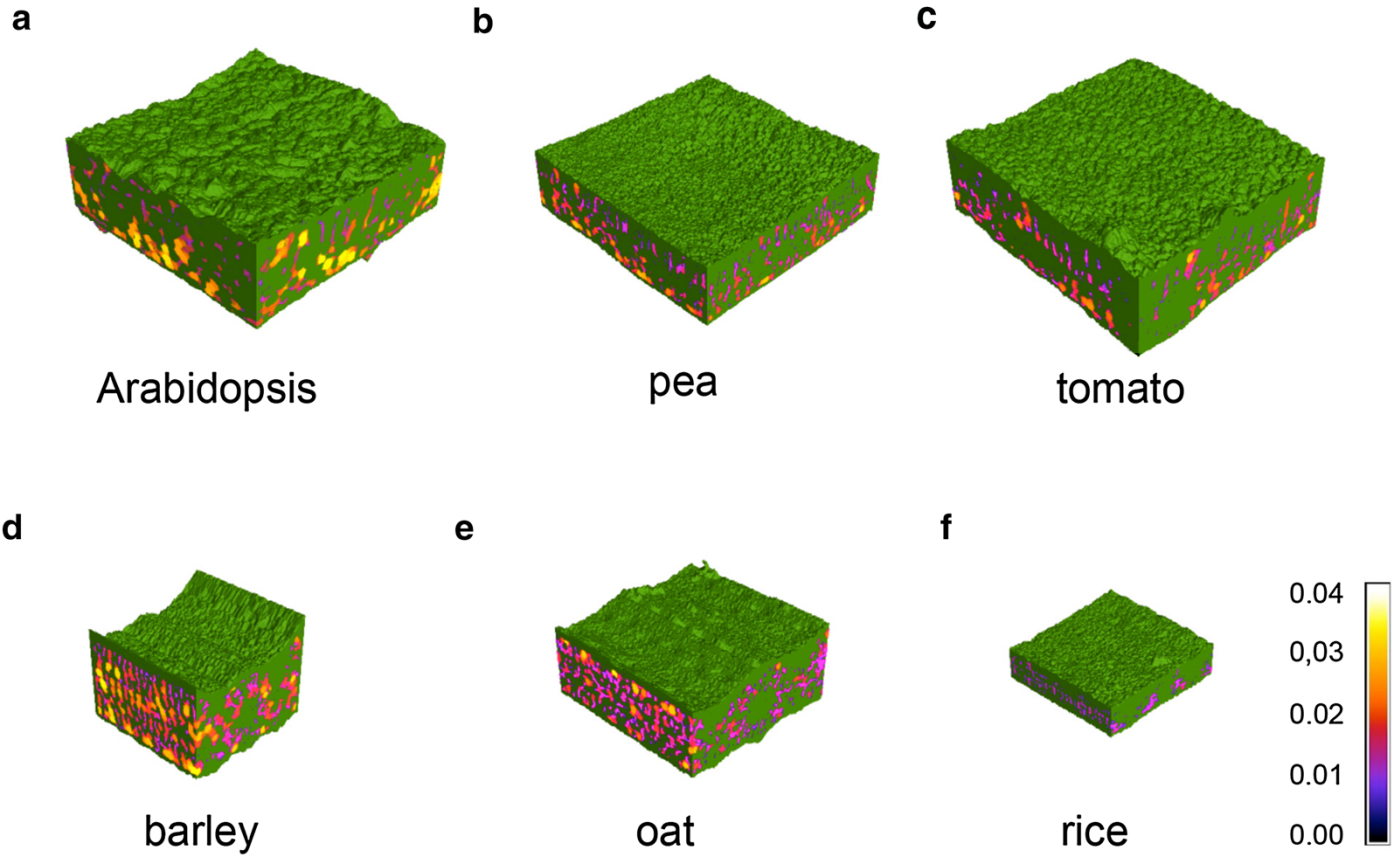
Representative 3D renderings from single microCT scans of leaf selections from three dicot species **a** *Arabidopsis thaliana*, **b** pea, **c** tomato and three monocot species, **d** barley, **e** oat and **f** rice, highlighting differences in leaf structure and air channel thickness. Leaf tissue is coloured green, while air channel thickness (diameter, mm) has been represented by a ‘heat map’ colour scale where hotter colours represent larger channel diameters. 3D renderings vary in size between species: the sizes correspond to the region of interest used for analysis. For the rice samples, three such areas were analysed per sample and averaged together

Using the described methods, X-ray microCT yielded high quality images and reproducible quantitative data from a variety of plant species including monocots and dicots. In the 3D reconstructions (Fig. 2), air channel size can easily be visualised using the ‘heat map’ colour scale of air channel diameter, in which channels with hotter colour (yellow or white) are the largest and cooler (blues) are the smallest. In Arabidopsis (Fig. 2a), the stereotypical dicot mesophyll can be seen clearly, with the largest pores in the abaxial spongy layer, and smaller pores in the adaxial palisade tissue. Rice (Fig. 2f) had the smallest air channels of the six species, with its airspace coloured entirely in pink and blue on the heat map scale. The 2D sections also allowed the measurement of leaf thickness. Rice showed the thinnest leaves with a thickness of 0.1 mm to oat with the thickest at 0.31 mm (pea = 0.21 mm, tomato = 0.23 mm, Arabidopsis = 0.26 mm and barley 0.29 mm).

**Fig. 3:**
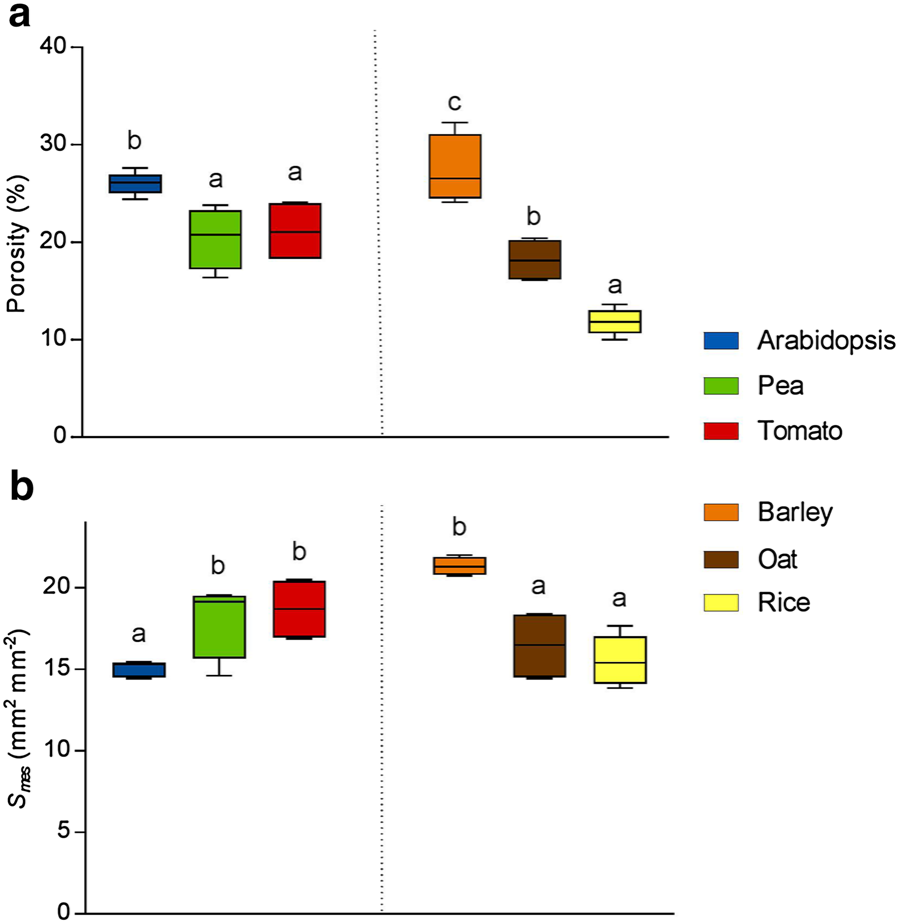
Quantitative analysis of leaf structures showed differences between species. **a** Leaf porosity values (%) for three monocot and three dicot species. **b** Surface area of the mesophyll cells exposed to intercellular airspaces (*S*_*mes*_) per unit leaf area (mm^2^ mm^−2^). N = 5–6 plants. One-way ANOVA followed by Tukey’s multiple comparisons test, with analyses conducted separately for monocots and for dicots (dicots F = 6.2, P = 0.02; monocots F = 16.9, P < 0.01). Boxes with a letter in common are not significantly different from one another at the 95% confidence level. Error bars represent standard error of the mean (SEM)

A number of biologically relevant parameters can be quantified from the 3D data (Additional file 1: Table S1). Here we focus on two of these considered to be important determinants of photosynthetic performance: leaf porosity (the proportion of leaf volume occupied by airspace) and *S*_*mes*_ (the surface area of mesophyll cells exposed to intercellular airspaces) (Fig. 3). Arabidopsis stands out among the surveyed dicots as the most porous, with a mean porosity of 26.0% ± 0.6 compared to 20.5% ± 1.6 (pea) and 21.1% ± 1.6 (tomato). Among the monocots, barely had the highest porosity (27.4% ± 1.8) and rice, the lowest (11.8% ± 0.6), with oat intermediate (18.2% ± 1.1) (See Additional file 3 for further example images of plant leaves used in the study).

The quantification of the surface area of mesophyll cells exposed to intercellular airspace (*S*_*mes*_) allows testing of established ideas about the importance of this factor in CO_2_ uptake. We calculated *S*_*mes*_ for each of the six species (Fig. 3b). The dicots with the highest mean values of *S*_*mes*_ were pea (18.1 ± 1.2 mm^2^ mm^−2^) and tomato (18.7 ± 1.0 mm^2^ mm^−2^), significantly higher than Arabidopsis, which had the lowest value of all six species (15.0 ± 0.2 mm^2^ mm^−2^). Barley had the greatest *S*_*mes*_ value (21.3 ± 0.3 mm^2^ mm^−2^), significantly higher than the other two monocots (oat 16.5 ± 1.1 and rice 15.5 ± 0.7 mm^2^ mm^−2^). Both porosity and *S*_*mes*_ measurements were highly reproducible between biological replicates, as demonstrated by the low standard error values across the range of species.

### Comparison of 2D and 3D quantification of rice leaf cellular architecture

**Fig. 4:**
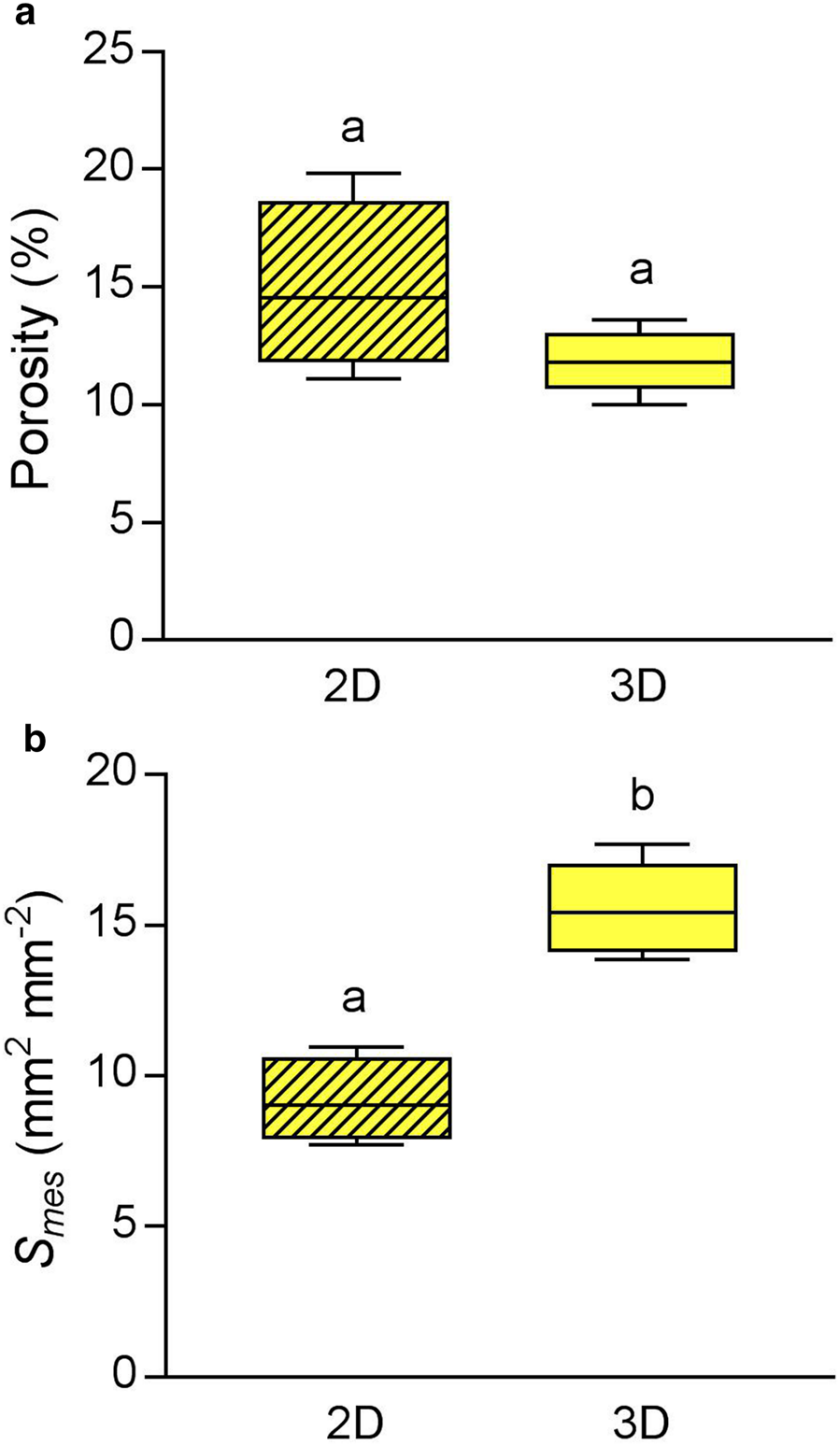
Comparison of 2D analysis of embedded sections of the same samples used for microCT-scanned rice leaf tissue estimates a similar porosity value to microCT (Unpaired t-test, t = 1.8, *df* = 7, P = 0.11) (**a**) but a lower value of *S*_*mes*_ (Unpaired t-test, t = 6.4, df = 7, P < 0.01) (**b**). Boxes with a letter in common are not significantly different from one another at the 95% confidence level. Error bars represent SEM

After microCT scanning, rice leaf discs were fixed, embedded and sectioned for analysis using an established 2D method (as described in [39]) to allow comparison with the 3D porosity and *S*_*mes*_ data (Fig. 4) obtained for the same samples. The porosity values from the 2D sectioning method and the 3D microCT method were not significantly different at the 95% confidence level (Unpaired t test, t = 1.8, *df* = 7, P = 0.11), although the spread of values was much lower in the microCT-based analysis. The calculation of *S*_*mes*_ from 2D sections was significantly lower than that from microCT data (Unpaired t-test, t = 6.4, *df* = 7, P < 0.01). This discrepancy (almost 50% higher values for *S*_*mes*_ calculated from microCT analysis than standard 2D analysis of sectioned material) was larger than that reported by Théroux-Rancourt et al. [12], whose estimations from microCT and the curvature correction factor method were typically within 10% of one another. This could be due to the relatively small number of 2D images (4–5 sections, at least a cell’s length apart) used to estimate the range of tissue structure through the leaf samples in our experiments, but nevertheless the difference in estimated mean values are striking.

### Structural variation within leaves

**Fig. 5:**
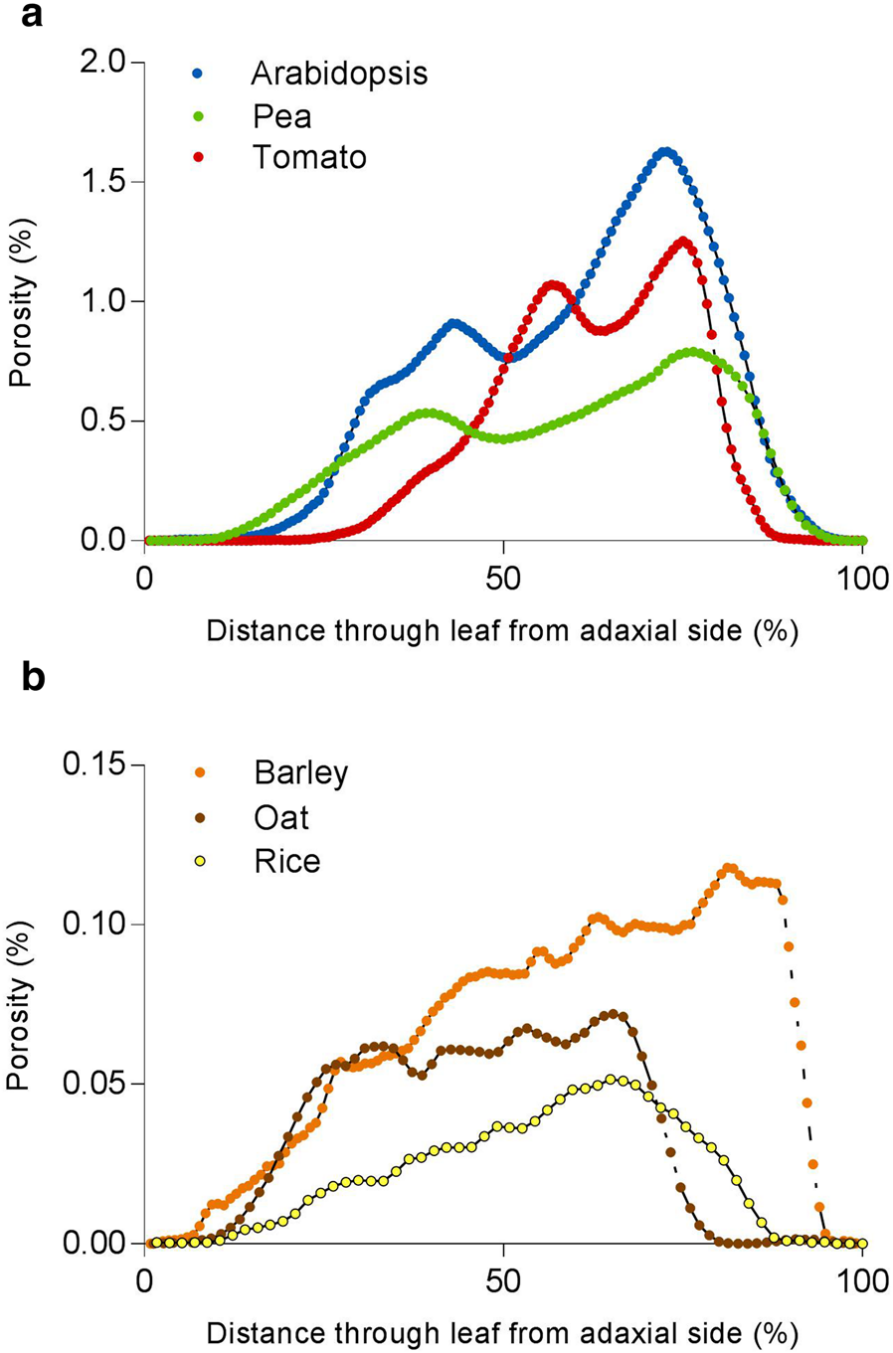
Porosity (%) values for each z-slice, plotted against distance through the leaf from adaxial to abaxial in one representative individual from each of three dicot species (**a**) and three monocot species (**b**)

The 3D data sets allow extraction of more detailed information about the spatial distribution of airspace than can be readily obtained using stereological approaches. The structural differences between monocot and dicot leaves are clearly displayed by plotting porosity against distance through the leaf (Fig. 5). In all six species, the region of very low porosity in the outer boundaries of the leaf corresponds to the densely packed epidermal cells, among which only stomatal pores create airspaces. The two distinct mesophyll layers typical of dicots can clearly be seen in Fig. 5a: the densely packed palisade tissue on the left side of the graph (adaxial side of leaf, low porosity), and the more open structure of the spongy mesophyll further right (abaxial side of leaf, high porosity). In monocot species (Fig. 5b) there is a much more gradual increase in porosity from the adaxial epidermis through the mesophyll, to the abaxial epidermis. Lower adaxial porosity in monocots results from the presence of large, densely packed bulliform cells on that side of the leaf, combined with the greater number and/or size of sub-stomatal cavities on the opposite, abaxial side of the leaf. Reflecting the overall mean porosity data shown in Fig. 4, Arabidopsis leaves displayed a higher porosity than the other two dicot species across the entire depth of the leaf, and barley porosity values were higher than the other two monocot species analysed at virtually all positions within the leaf. While oat leaves displayed a very symmetrical distribution of air space across the adaxial/abaxial axis, in barley there was a clear asymmetry, with the abaxial side of the leaf generally having higher porosity, with rice showing an intermediate distribution of airspace.

## Discussion

Leaf cellular architecture is known to play an important role in photosynthesis. With the development of more advanced equipment, software and protocols, such as those described here, it is now possible to visualise leaf internal air channels at sub-micron resolution, and to quantify biologically relevant aspects of the air channel network. This method allows rapid imaging of live tissue samples at high resolution. Our previous work has successfully employed this technique for the characterisation of Arabidopsis mutants [34, 35] and here we demonstrate that it can be successfully applied to a wider range of plant species.

The relatively rapid scan time is a key advantage of this method, as it allows the use of live tissue. Crucially, the leaf structure does not change through desiccation during the course of such a rapid scan providing it is adequately supported by the radio opaque polystyrene foam. Fast scanning has the additional advantages of allowing a higher throughput rate than other microCT protocols, and mitigating problems of X-ray induced damage that could occur with prolonged or repeated scanning [23]. The use of live tissue minimises preparation time and removes the risk of artefacts that could be introduced by fixation and staining. Established stereological methods generally use embedded tissue, which risks structural changes during fixation or dehydration stages of the embedding process. Furthermore, the much lower tissue coverage in 2D approaches compared to tomography tends to lead to underestimation of *S*_*mes*_, as demonstrated by Théroux-Rancourt et al. [12] who sampled 2D slices from their 3D image stacks for a robust comparison. Our comparison of 2D and 3D analysis of identical rice leaf discs confirmed previous reports that 2D approaches lead to lower *S*_*mes*_ estimates. It should be stressed that during the image analysis procedure careful testing of the most appropriate automated threshold algorithm must be investigated. An algorithm suitable for one plant species may not be appropriate for another and lead to under or over estimations of porosity and Smes. The very low standard errors for each group of biological replicates suggest that our protocol for microCT analysis is robust and reliable within plant species where the same threshold algorithm was applied.

While the tissue is live at the time of scanning, our method does require destructive sampling. Repeated scans of the same sample after a treatment or over the course of development are therefore not possible. MicroCT scanners are available to scan larger samples, but this comes with a trade-off in resolution that would prevent accurate extraction of such small structures as the leaf airspace network [40]. Furthermore, holding the sample sufficiently stationary to obtain a clear image without detachment from the plant presents a challenge. Excision of leaf discs does result in an area of collapsed tissue around the edge of the sample, but this can be excluded when selecting the ROI for analysis. The disc must be handled with great care during mounting to ensure that no further damage occurs. We make the assumption that any wound response in the tissue does not result in a change in the structure of the center of the disc during the timescale of the scan.

Current limitations on the achievable contrast and resolution of live tissue with lab-based microCT equipment (X-ray absorption based) prevent individual cells from being distinguished in the images. Finding a way to resolve cellular detail would be a challenging but useful target for the future, offering insights into the developmental processes that lead to the formation of the airspace network. Scanning at even greater resolution can produce stacks in which individual cells can be seen in live tissue, but this has only been demonstrated with synchrotron-based microCT to date [25, 41, 42]. Alternatively, increasing the contrast between cells and tissues, or boosting the contrast of the cell outlines (cell wall/cell membrane) using phase contrast techniques have demonstrated improved edge detection of cellular features in plant roots [43]. Dhondt et al. [23] used iodine as a contrast agent to obtain detail at the individual cell level, but this required more extensive tissue preparation and a much slower scan time. Even if appropriate contrast agents were available for use with live samples, infiltrating them through the full tissue depth would be challenging. Until such a method is available, classical histological techniques will remain useful to complement the microCT data. Recently, combined microCT and histological approaches have provided valuable insights in biomedical studies [44, 45]. The adoption of similar approaches to plants may enable sub-cellular structures to be revealed, such as plastid size and position, which are highly relevant for understanding photosynthesis but cannot be obtained by tomography alone.

Selection of the ROI for computational analysis is critical for obtaining realistic and comparable numerical data. Firstly, damaged tissue areas must be avoided. It is also desirable to avoid veins as far as possible, although this brings a trade-off with ROI size. In these analyses we sought to use the largest ROI possible without inclusion of major veins. In the rice samples, the veins were sufficiently close together that we took multiple ROIs from each scan for the analysis to sample a sufficiently large vein-free area. Taking multiple ROIs per sample is a more labour-intensive approach as each region must be image processed separately. However, a further advantage of smaller ROI is that there is a greater possibility of selecting a flat region of leaf, which in turn makes the separation of distinct leaf layers as sets of z-slices more feasible, such as palisade and spongy mesophyll in dicots. Smaller ROIs also make it possible to avoid other structures, such as large trichomes, which might skew airspace quantification. While structures such as veins and trichomes are currently a complication that we have tried to avoid in our analysis, they are part of the true leaf structure. As our models of leaf development and of gas exchange networks advance, the inclusion of these features in ROIs may become useful and informative.

The image processing workflow presented here allows for the largely automated calculation of many morphological descriptors of the extent and spatial patterning of the leaf airspace network. However, some stages of the analysis still require manual verification by the operator, which are slower and more subjective than the automated steps. Generating the mask to define the tissue volume (as distinct from background and packing elements) is a semi-automated process, but in some species required extensive manual input. In Arabidopsis, for example, masking areas with trichomes requires some manual input if these leaf hairs are to be excluded so as not to affect the quantitative data. Furthermore, the density of some areas of the polystyrene packing discs is similar to that of the Arabidopsis cells (especially if the polystyrene has been compressed), requiring that these regions adjoining the tissue be manually removed from the masks. Defining the automated threshold value for image binarisation is also a manual step, and therefore somewhat subjective. Unfortunately, the availability of suitable ground truthing techniques to support the decision-making process is sadly lacking and therefore the informed ‘expert’ assessment of a trained user is required on a species by species basis. After this initial decision has been reviewed an automated analysis routine can be implemented.

The quantification and spatial mapping of leaf airspace allows us to probe the relationship between structure and function in the leaf by measuring gas exchange in plant lines that vary in their cellular architecture. Dorca-Fornell et al. [34] reported that, in Arabidopsis plants with altered expression of a cell cycle regulator, an increase in leaf porosity led to a significant increase in stomatal conductance but, perhaps surprisingly, no related increase in carbon assimilation. In contrast, demonstrated a positive correlation between mesophyll cell density and photosynthetic capacity among Arabidopsis mutants with cell cycle gene expression manipulated in targeted tissue layers. These results suggest a complex relationship between leaf structure and photosynthesis, which may involve effects of both gas exchange and light attenuation. Network analysis of the air channels within the leaf may contribute to modelling of airspace arrangements that might increase mesophyll conductance, allowing more effective gas exchange.

In addition to investigating the effects of leaf structure on gas exchange, these morphological data can be used to investigate relationships between leaf structural parameters. We might expect, for example, that porosity and *S*_*mes*_ would be inter-dependent, but our data suggest this relationship is not so simple. This becomes a question of cell packing which will be influenced by a range of factors, such as the size and shape of mesophyll cells and the local control of cell separation. For example, increasing the extent of lobing in rice mesophyll cells could elevate *S*_*mes*_ without greatly changing porosity. Understanding how to manipulate the development of such elements of leaf structure (and having a robust means of quantifying the output structural parameters) is essential if we are to use such information to manipulate leaf structure with a view to optimising photosynthetic performance.

## Conclusions

The method presented here allows for the high resolution imaging of leaf intercellular airspace networks by lab-based microCT, and the largely-automated, quantitative description of those networks. These data can be used to investigate both developmental phenomena, such as the inter-relation of structural parameters, and physiological questions about the effect of leaf structure on gas exchange and photosynthesis. Technical advances in microCT imaging (e.g. improved contrast X-ray detectors with higher sensitivity) may, in the future, offer possibilities for gathering even more detailed information on leaf structure from live tissue, such as resolving individual cells. However, data at the level of detail that can currently be achieved already offer much unexplored potential for testing established ideas and developing new hypotheses to establish which leaf structural features are the most important for photosynthesis. This understanding, combined with knowledge of leaf developmental genetics, could facilitate the re-engineering of the leaf to enhance plant productivity.

## Additional files

**Additional file 1: Table S1.** Measurements that can be extracted from microCT data, with their units and definitions, and the plugins required for their calculation.

**Additional file 2: Figure S1.** 2D image analysis workflow of microscope images.

**Additional file 3: Figure S2.** 2D and 3D representative microCT images of plant leaves used in this study.
