# Supplementary material for: Investigating the microstructure of plant leaves in 3D with lab-based X-ray computed tomography
Source: Plant Methods. 2018 Nov 12;14:99. doi: 10.1186/s13007-018-0367-7 (PMC6231253; doi:10.1186/s13007-018-0367-7)
Supplement: Supplementary file 1 — Additional file 1: Table S1. Measurements that can be extracted from microCT data, with their units and definitions, and the plugins required for their calculation. [file 13007_2018_367_MOESM1_ESM.docx]

**Supplementary information**

**Table S1.** Measurements that can be extracted from microCT data, with their units and definitions, and the plugins required for their calculation.

| **Parameter** | **Units** | **Definition** | **Plugin** |
| --- | --- | --- | --- |
| Porosity | **%** | The proportion of the tissue volume occupied by airspace | ImageJ Analyse particles |
| *S_mes_* | mm^2^ mm^-2^ | The surface area of mesophyll cells exposed to intercellular airspace per unit leaf area | ImageJ Analyse particles |
| Channel density | Channels per mm^2^ | The number of individual air channels per unit area of z-slice | ImageJ Analyse particles |
| Mean air channel diameter | mm | Based on a measure of mean local thickness of the channel diameter in 3D | BoneJ Thickness |
| Max. air channel diameter | mm | Based on the maximum local thickness of the channel in 3D | BoneJ Thickness |
| 3D channel density | Channels mm^-3^ | The number of air channels in 3D | BoneJ Connectivity |
| Degree of connectivity | Channels mm^-3^ | Channel number per mm^3^ | BoneJ Connectivity |
| Euler characteristic |  | Describes the topology of the 3D shape of the channels | BoneJ Connectivity |
| Number of channels | - | Total number of channels | BoneJ Analyse Skeleton |
| Mean channel length | mm | The average distance between nodes in the skeletonised network | BoneJ Analyse Skeleton |
| Max channel length | mm | The maximum distance between nodes in the skeletonised network | BoneJ Analyse Skeleton |
| Tortuosity | - | Measures channel network tortuosity in 3D. Can be useful to understand gas diffusion. | BoneJ Analyse Skeleton |

**Supplementary Methods**

**Airspace network analysis**

The number of connections present within the selected leaf volume, the connection density and the degree of connectivity, were calculated using the ‘Connectivity’ function in BoneJ. This function assumes that there is only one foreground particle and it contains no cavities (inclusions). Therefore, prior to connectivity analysis, binary image stacks were inverted to make the intercellular airspace the subject of the images (foreground), then run through the ‘Purify’ function in BoneJ, which eliminates all but the largest foreground and background particles. Connection density (Conn. mm^-3^) was calculated by dividing the number of connections present within the sampled leaf volume by the material mask volume, rather than using the value directly calculated by the ‘Connectivity’ function. The connectivity of each leaf disc was expressed as its Euler Characteristic, which was directly calculated by the ‘Connectivity’ function, using a voxel neighbourhood algorithm.

The structural architecture and composition of the intercellular airspace present within each leaf disc was analysed using the ‘Analyze Skeleton’ function in BoneJ. Prior to this analysis inverted binary image stacks were skeletonized using a 3D thinning algorithm [41] embedded in the ‘Skeletonise 3D’ function in BoneJ. The number of channels (branches) present within the volume, mean channel length (mm), max channel length (mm), Euclidean distance (mm) and channel density (channels mm^-3^) were all directly calculated by the ‘Analyze Skeleton’ function. Channel tortuosity (degree of twistedness) was calculated using Equation 5.

$Tortuosity= \frac{D_{\mathrm{euc}}}{L_{b}}$ Equation 5.

Where, *D*_euc_ is the Euclidean distance (mm) and *L*_b_ is the branch length (mm).
