# Supplementary material for: Investigating the microstructure of plant leaves in 3D with lab-based X-ray computed tomography
Source: Plant Methods. 2018 Nov 12;14:99. doi: 10.1186/s13007-018-0367-7 (PMC6231253; doi:10.1186/s13007-018-0367-7)
Supplement: Supplementary file 2 — Additional file 2: Figure S1. 2D image analysis workflow of microscope images. [file 13007_2018_367_MOESM2_ESM.pdf]

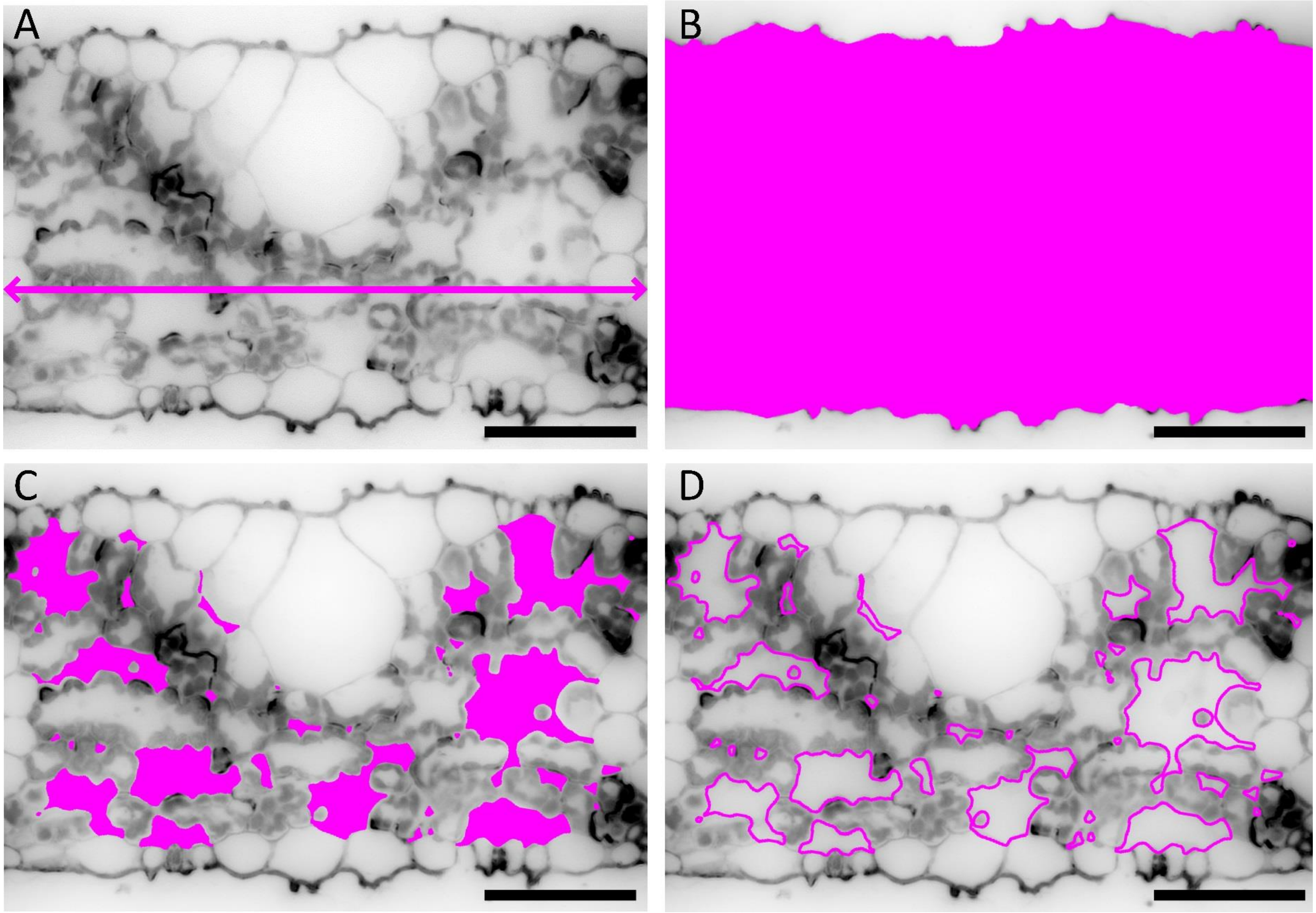

Figure S1. 2D image analysis workflow. (A) Measurement of section width for S_{mes} 3D estimation (S_{mes} = airspace perimeter/section width x F, where F = 1.42). (B) Masking the total section area. (C) Masking the intercellular airspaces. (D) Measuring the total airspace perimeter in the 2D section. Scale bar = 50µm.
