# Supplementary material for: Investigating the microstructure of plant leaves in 3D with lab-based X-ray computed tomography
Source: Plant Methods. 2018 Nov 12;14:99. doi: 10.1186/s13007-018-0367-7 (PMC6231253; doi:10.1186/s13007-018-0367-7)
Supplement: Supplementary file 3 — Additional file 3: Figure S2. 2D and 3D representative microCT images of plant leaves used in this study. [file 13007_2018_367_MOESM3_ESM.pdf]

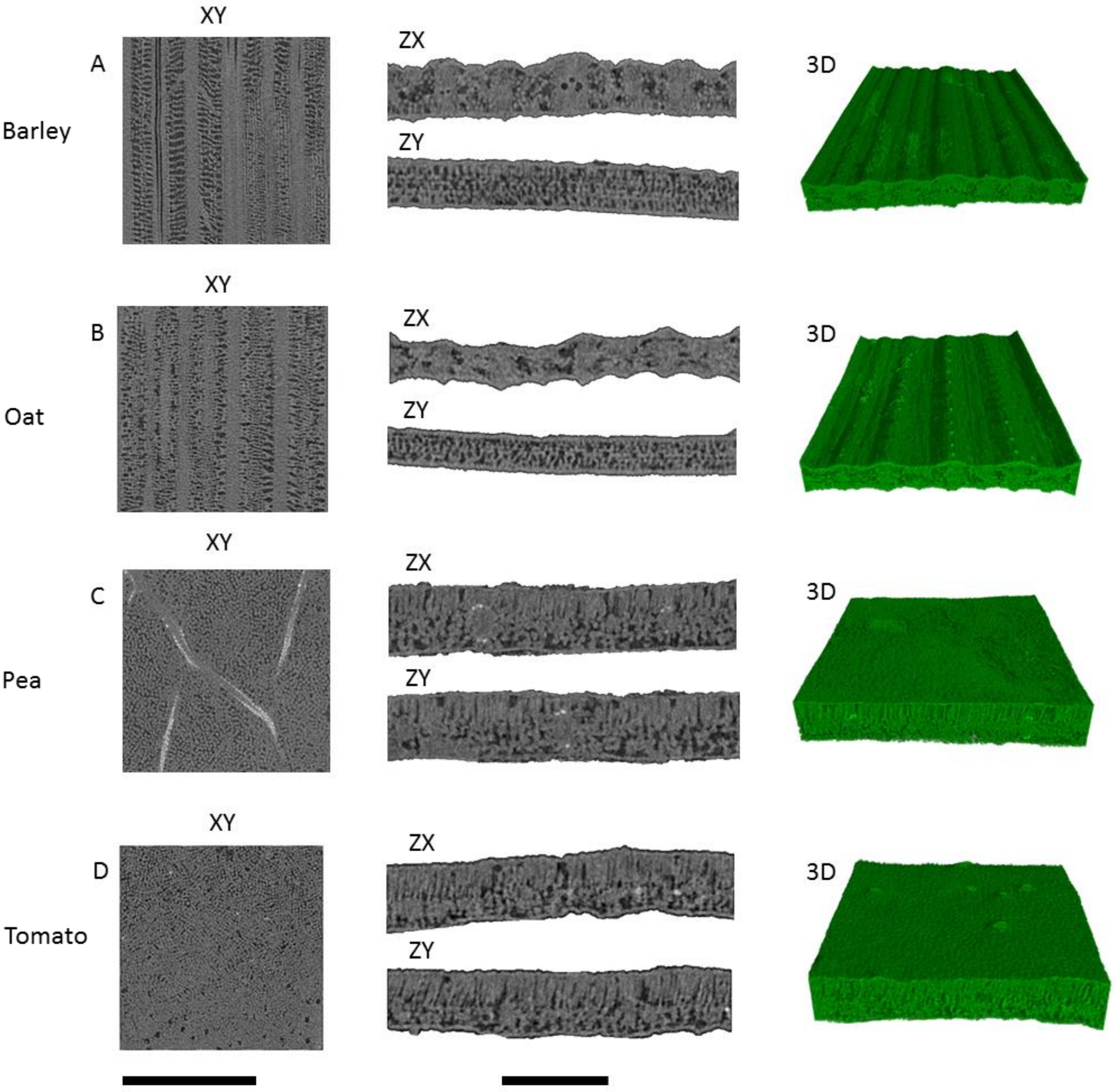

Figure S2. 2D and 3D representative microCT images of (A) barley, (B) oat, (C) pea and (D) tomato. 2D images show XY (top down), ZX (front), ZY (right) slices through the 3D data. Darker colours in the images relate to lower density material (e.g. air space) with higher density material showing brighter colour intensity (e.g. veins of the pea). Scale bars are 0.85 mm for XY images and 0.4 mm for the ZX and ZY images.
